# Adenoid cystic carcinoma: current therapy and potential therapeutic advances based on genomic profiling

**DOI:** 10.18632/oncotarget.5076

**Published:** 2015-08-21

**Authors:** Young Kwang Chae, Su Yun Chung, Andrew A. Davis, Benedito A. Carneiro, Sunandana Chandra, Jason Kaplan, Aparna Kalyan, Francis J. Giles

**Affiliations:** ^1^ Northwestern Medicine Developmental Therapeutics Institute, Northwestern University Feinberg School of Medicine, Chicago, IL, USA; ^2^ Robert H. Lurie Comprehensive Cancer Center of Northwestern University, Northwestern University Feinberg School of Medicine, Chicago, IL, USA; ^3^ Department of Medicine, Northwestern University Feinberg School of Medicine, Chicago, IL, USA

**Keywords:** adenoid cystic carcinoma, targeted therapy, genetics, immunotherapy

## Abstract

Adenoid cystic carcinoma (ACC) is a rare cancer with high potential for recurrence and metastasis. Efficacy of current treatment options, particularly for advanced disease, is very limited. Recent whole genome and exome sequencing has dramatically improved our understanding of ACC pathogenesis. A balanced translocation resulting in the *MYB-NFIB* fusion gene appears to be a fundamental signature of ACC. In addition, sequencing has identified a number of other driver genes mutated in downstream pathways common to other well-studied cancers. Overexpression of oncogenic proteins involved in cell growth, adhesion, cell cycle regulation, and angiogenesis are also present in ACC. Collectively, studies have identified genes and proteins for targeted, mechanism-based, therapies based on tumor phenotypes, as opposed to nonspecific cytotoxic agents. In addition, although few studies in ACC currently exist, immunotherapy may also hold promise. Better genetic understanding will enable treatment with novel targeted agents and initial exploration of immune-based therapies with the goal of improving outcomes for patients with ACC.

## EPIDEMIOLOGY OF ADENOID CYSTIC CARCINOMA

Adenoid cystic carcinoma (ACC) is a rare cancer most often occurring in the salivary glands. For head and neck ACC, the age-adjusted incidence rate is 4.5 cases per 100,000 individuals, occurring with a slight female predominance (60% vs. 40% in males) [1, 2]. The cancer can also arise in other locations including the breast, skin, respiratory system, and reproductive organs [3]. The mean age at diagnosis of head and neck ACC is approximately 57 years, which varies based on primary cancer site [2, 3]. ACC is characterized by an indolent clinical course and has a tendency for delayed recurrence and metastasis after initial treatment [4]. Five, ten, and fifteen-year survival rates after surgical resection have been reported as 77.3%, 59.6%, and 44.9%, respectively, with almost half of patients dying from ACC, as opposed to other causes, at long-term follow-up [5]. Because of its rarity and distinctive clinical features, the pathophysiology of ACC remains understudied, resulting in fewer evidence-based therapies compared to other cancers.

### Histopathology of adenoid cystic carcinoma

ACC is subdivided into 3 histological groups based on solid components of the tumor including cribriform, tubular, and solid. Cribriform and tubular ACCs usually exhibit a more indolent course, whereas the solid subtype is associated with worse prognosis [6, 7]. Szanto *et al*. demonstrated that grade III ACC, which displayed marked predominance of the solid portion, resulted in larger tumors that recurred more frequently and had lower cumulative survival rate compared with the other groups [8]. Solid histology has been associated with higher somatic mutation frequency, as well as particular chromosomal and genetic markers including loss of 14q and alterations in the PI3K pathway [9, 10]. In addition, several factors have been associated with disease recurrence including solid type histology, higher histological grade, pathologic stage, and tumor size [11].

ACC consists of two different cell types: inner luminal epithelial cells and outer myoepithelial cells. Previous studies have demonstrated that each cell line tends to express different molecular biomarkers. In particular, epithelial cells express c-kit, cox-2 and Bcl-2, while myoepithelial cells express EGFR and MYB. This discrepancy is likely important in understanding the heterogeneity of ACC, including tumor etiology and various responses to current treatments [12–14].

## MOLECULAR PATHOLOGY OF ADENOID CYSTIC CARCINOMA

### Genetic alterations related to myeloblastosis oncogene (MYB)

Multiple studies have been conducted to discover genetic mutations and biomarkers specific for ACC. Among them, a balanced translocation of the v-myb avian myeloblastosis viral oncogene homolog-nuclear factor I/B (*MYB-NFIB*) is considered to be a signature molecular event of ACC oncogenesis. A 2009 study by Persson et al., reported recurrent episodes of *MYB-NFIB* fusion genes in ACC specimens [15]. The chromosomal translocation t(6; 9)(q22–23; p23–24) produced chimeric transcripts containing *MYB* and *NFIB* and caused deregulation of the *MYB* gene. As a result, genes targeted by *MYB* were activated constitutively, resulting in downstream dysregulation of critical cellular mechanisms involved in apoptosis, cell adhesion, and cell cycle regulation [15].

Although another study performed on 102 ACC tumors also showed a significant *MYB-NFIB* gene fusion rate (52.8%, detected by FISH), the fusion transcripts were detected at lower rates (38.2%) [16]. Another group compared ACC with other salivary gland tumors and non-salivary gland cancers and focused on *MYB* expression. In this study, the *MYB-NFIB* balanced translocation was identified in 49% of ACC tissues. On the other hand, a portion of specimens demonstrated abnormal *MYB* FISH pattern without *NFIB* involvement [17]. Similarly, overexpression of *MYB* was previously identified in fusion-negative ACC samples [13]. These results potentially implicate other genetic mechanisms that could trigger malignant transformation besides the *MYB-NFIB* fusion event, such as an isolated function of the *MYB* gene. *MYB*, located on 6q22–23, is a crucial gene in cell differentiation. This abnormality has been reported in various malignancies, including some types of leukemia and solid tumors such as melanoma [12, 18]. As a transcription factor, *MYB* is known to modulate multiple genetic downstream targets involved in oncogenesis, such as cox-2, c-kit, Bcl-2 and BclX [12].

The importance of *MYB* in the pathogenesis of ACC was further demonstrated by a recent analysis of whole exome sequencing. In this study, the *MYB-NFIB* fusion gene was confirmed in 57% of the total ACC specimens, in accordance with prior studies [9, 16, 17]. In addition to the fusion gene, mutations in each of the *MYB* and *NFIB* genes were detected. Splice site and coding mutations were observed in *MYB*, and several genes related to the *MYB* pathway such as *MGA, MYCBP2 and MCM4* were also mutated. The finding of two truncating mutations and four homozygous deletions in the *NFIB* gene also suggested a possible independent role of *NFIB* in carcinogenesis of ACC [9]. In terms of prognosis, one study suggested that high *MYB* exon expression was associated with poor patient survival, independent of the solid phenotype, with the combination of high *MYB* expression and solid histology leading to the worse patient survival [16]. However, another study reported no association between *MYB-NFIB* positive tumors and longer disease-free or overall survival [19]. Clearly, further studies are needed to better associate clinical outcomes with molecular findings, but the frequency of the *MYB-NFIB* fusion transcript reinforces a clear clinical link for future therapeutic interventions.

### Newly detected genetic mutations through genome sequencing

The recent whole exome and genome sequencing of ACC has led to new compelling discoveries. Ho *et al*. analyzed sequences of 60 ACC tumors paired with normal DNA samples. The goal was to discover driver genes, which revealed genomic alterations in the pathways associated with *MYB/MYC*, chromatin remodeling, tyrosine kinase signaling, and DNA damage/checkpoint signaling [9].

Interestingly, a significant portion of ACC cells did not exhibit *MYB* involvement, which implicated new potential mechanisms in disease pathogenesis [17]. In line with this, multiple mutations in chromatin regulators were found in 35% of ACC tumors by this genomic sequencing [9]. For example, *SMARCA2* was one of the genes found to be mutated in ACC. This gene participates in gene transcription by encoding a DNA dependent ATPase of the mammalian SWI/SNF chromatin remodeling complex [20]. Though mutations in *SMARCA2* are reported in a rare developmental disorder [21], the gene has not yet been directly related to any specific cancer [9, 22]. This may suggest that mutations in *SMARCA2* could be involved in ACC tumorigenesis. Other mutated genes associated with gene transcription include *SMARCE1*, *ATRX* and *ARID1A* (Table 1). However, the importance of these mutations in ACC remains undefined.

**Table 1:** Genes with molecular aberrations classified by their involved pathways

Pathways	Ho *et al*. [9]	Stephens *et al.* [22]
MYB/MYC	*MYB, NFIB, MYBL1, MYCN, MYCBP2, MGA, MCM4*	*MYB*
Epigenetic modification (Chromatin remodeling)	*SMARCA2, SMARCE1, ARID1A, ATRX, SRCAP, CREBBP, KDM6A, KDM6B, JMJD1C, EP300, ARID4B, ARID5B, BRD1, MLL3, FTSDJ1, HIST1H2AL, HIST1H1E, MORF4L1, KAT6A, KANSL1, SETD2, NSD1, BCOR, BCORL1*	CREBBP, SF3B1, ARD1A, SPEN*, EP300, KDM6A, MLL3, ARID1B, SMARCA2, CHD2, BRD2, ARID5B, KDM5A
DNA damage/checkpoint	*TP53, UHRF1, TXNIP, ATM, BRCA1, DCLRE1A, PRKDC, SMC1A, TLK1*	*ATM, CDKN2A*
FGF/IGF/PI3K signaling	*PI3KA, PTEN, FOXO3, FGF16, FGFR4, IGFBP2, ILR17RD, INSRR, MAGI1, MAGI2, ERBB2IP, HRAS, MAPK2*	*FGFR2, TSC1, PI3KA*
NOTCH signaling	*NOTCH1, FOXP2, DTX4, FBXW7, CNTN6, MAML3*	*NOTCH1, NOTCH2*
Others	*RYR3, RYR2, PTPRG, PTPRH, PTPRJ, PTPRK, HSPG2, IDH1, NTNG1, SEMA3G, SEMA5A, FAT3, FAT4*	*SUFU, CYLD*

* *SPEN* also interacts with NOTCH signaling pathway, but it is classified here as it is basically a transcriptional regulator.

Histone-related genes/proteins also take part in chromatin remodeling. Coordination of histone acetyltransferase/deacetylase regulates gene transcription by altering chromatin structure [23–25]. Based on this mechanism, histone deacetylase (HDAC) has become a novel target of anti-cancer agents [24–26]. Genes linked to HDAC activity (e.g., *ARID4B*) were previously shown to be mutated in ACC cell lines [9]. Other genes related to histone activity such as *CREBBP*, *EP300, ARID5B, KDM6A*, and *BRD1* were also mutated in ACC [9, 22].

Various signaling cascades are essential for cancer cells to survive and grow. The PI3K/Akt/mTOR pathway is one of them. This pathway regulates cell survival and growth and is upregulated in many cancers. Involved in the PI3K pathway, *PI3KA* is a driver oncogene mutated in numerous cancers [27]. This oncogene was identified in a subset of ACCs as well [9, 22]. Other mutated genes associated with this pathway include *PTEN, FGF16, FGFR4 and ILR17RD* [9]. The PKA pathway is also critical for cancer cell survival and was found to have mutations in its related genes in 27% of samples [9]. This pathway increases cell survival in the stressful condition via cAMP-dependent activation [28] or may induce cell death by interacting with Bcl-2 in other situations [29]. Recurrent mutations in *RYR2* and *RYR3* were detected in this pathway, and other associated genes, *PTPRG, PTPRH, PTPRJ* and *PTPRK*, were also mutated in ACC [9].

Errors in the DNA repair system cause genomic instability, which may be associated with malignant transformation of cells. Mutations in genes associated with DNA repair are frequently found in familial cancer syndromes, such as hereditary breast-ovarian cancer syndrome (HBOC), hereditary non-polyposis colorectal cancer (HNPCC, also called Lynch syndrome) and Li-Fraumeni syndrome [30, 31]. These mutations were also reported in non-hereditary cancers [30, 32]. Some of these mutations have also been noted in ACC [33]. Whole genome sequencing revealed many mutated genes involved in the DNA damage signaling pathway with a 27% prevalence of the sample cohort. The mutated genes include *BRCA1, ATM, TXNIP, PRKDC* and *TP53* [9]. *TP53* is a tumor suppressor gene, which regulates cell death and DNA repair. It is the most frequently mutated gene in cancers, occurring in up to 75% of invasive cancers [34]. Somatic mutations in *TP53*, as well as related genes (e.g. *UHRF1*), were identified in ACC [9, 35]. In addition, a recent study demonstrated that down-regulation of p53 induced increased perineural invasion activity of ACC tumors, *in vitro* [36].

Alterations in the NOTCH signaling pathway, which is involved in cell differentiation, is observed in many cancers, including ACC [9, 22, 37]. Somatic mutations of both *NOTCH1* and *NOTCH2* have been reported [22]. Another study revealed up-regulation of NOTCH1 in ACC tissue compared with normal salivary gland, and this finding became much pronounced in metastatic/recurrent ACC tissue [38]. *SPEN*, another related gene regulating NOTCH signaling, has been found to have somatic mutations in solid type ACC [22]. Working as a transcriptional repressor, SPEN is a downstream effector of NOTCH signaling and this mutation was confirmed in high frequency [22, 37]. Another study confirmed that *SPEN* is located on 1q36, which was frequently deleted in ACC. Loss of this region also correlated with poor prognosis [39].

**Figure 1:**
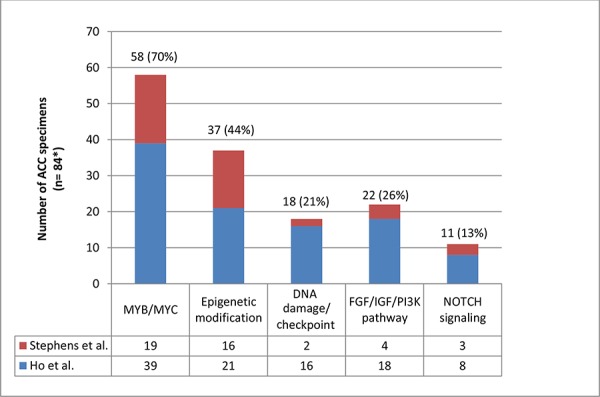
Distribution of molecular aberrations^+^ in 5 different pathways among patients with adenoid cystic carcinoma ^+^Molecular aberrations: somatic mutations, structural variants and deletions/focal amplifications [9]. *A sum of 60 samples from Ho *et al*. and 24 samples from Stephens *et al*. [9, 22]. More than one aberration may exist in a single specimen. Multiple aberrations in the same category in one specimen were considered as 1. For example, a specimen which harbors mutations in *EP300* and *BRD2* will be counted 1 time because those mutations belong to the same category of epigenetic modification. On the other hand, if a sample harbors 2 different categories of aberrations, this will be counted 2 times, each one representing a separate pathway.

There were two recent studies that showed the whole genomic landscape of ACC. One study classified genomic aberrations into 5 major pathways [9]. In this review, we applied the same principle to another article and combined the data as shown in Figure 1 and Table 1 [9, 22]. The total number of specimens was 84 (*n* = 60 from [9] and *n* = 24 from [22]). Of note, 70% of ACC samples (58 of 84) were found to have genetic alterations in the *MYB/MYC* pathway, indicating that changes in this pathway are crucial in ACC pathogenesis. The second most frequently mutated pathway was involved in chromatin remodeling (epigenetic modification), a pathway that includes multiple histone related proteins, and was altered in 44% of samples (37 of 84). The frequency of genetic changes in the other pathways is displayed in Figure 1. In addition, one recent genome wide association study looked at 98 cases of ACC. Although no significance was found on this subgroup analysis, a larger sample size would be beneficial as SNPs that approached significance for ACC were significant for another type of salivary gland carcinoma (mucoepidermoid carcinoma) [40].

### Proteins overexpressed in adenoid cystic carcinoma

Identifying biological markers in ACC is crucial for potential diagnosis and targeted therapy for patients. In this regard, effort to detect particular proteins in cancer cells has been made as one tool to facilitate the development of new diagnostic tools and targeted agents.

C-kit is a receptor tyrosine kinase which plays a pivotal role in cell signaling and eventually controls cell differentiation and growth [41]. Prior studies revealed expression of c-kit in many types of cancers such as acute myeloblastic leukemia, gastrointestinal stromal tumor, melanoma and even in rare cancers [42–45]. In ACC, previous studies have shown that c-kit is overexpressed primarily in epithelial cells [13, 46–48]. In a series of patients with ACC of the breast, 100% expressed c-kit and all expressed triple negative phenotype (ER-, PR-, HER2-), suggesting a unique exception to the typically aggressive triple negative tumors [49]. Based on these findings, targeted agents for c-kit have been explored in patients with ACC [50–54].

Additional studies have demonstrated that EGFR, a transmembrane glycoprotein receptor which activates multiple signal transduction pathways and ultimately regulates cell differentiation, survival and proliferation, is overexpressed in myoepithelial ACC cells [13, 48]. According to one study, EGFR positivity was reported in 56% (27 of 48) of ACC patients [48]. Similar to c-kit, EGFR overexpression was found in various cancers such as colorectal cancer, lung cancer, and head and neck cancers [55–57]. Multiple targeted agents aimed at EGFR or its tyrosine kinase are currently available. Therefore, these agents are potential therapeutic options for patients with ACC who overexpress EGFR [58].

Proteins working as transcription factors are also overexpressed in ACC. For example, members of the SOX family, such as SOX 4 and SOX10, are overexpressed in ACC [59, 60]. Upregulation of both proteins was also identified in breast cancer [60, 61], indicating that they are not specific for ACC. The RUNX3 protein is another transcription factor that was inversely correlated with tumor progression and worse prognosis in ACC possibly because *RUNX3* is a known tumor suppressor gene [62].

Angiogenesis plays an important role in cancer development and metastasis. Molecules involved in this process, such as VEGF, iNOS and NF-κB were noted to be highly expressed in ACC cells as compared to normal salivary gland cells [63, 64]. VEGF, c-kit, and EGFR were not correlated with ACC recurrence of prognosis in one study [48]. Regardless, agents targeting these molecules should be further explored for treatment of ACC.

In addition, recent studies have shown that FABP7 (Fatty acid binding protein 7) and AQP1 (Aquaporin 1) tend to be overexpressed in ACC cell lines. Therefore, these proteins are also potential biomarkers and therapeutic targets in ACC [65–68]. Overexpression of Bmi-1, which is implicated in the tumorigenesis of various malignancies and plays a role in cell proliferation, migration, and invasion, was correlated with clinical stage and prognosis of ACC in another study [69]. Similarly, overexpression of Pim-1 was associated with advanced stage and tumor aggressiveness in ACC, along with low level of RUNX3 [70].

Several studies have evaluated overexpression of HER2 in ACC. These studies have demonstrated considerable variability in HER2 overexpression ranging from 0–58% in patients with ACC [71]. Expression is highly dependent on subtype of salivary gland carcinoma. For example, one study reported that while 17% (23 of 137) of patients with salivary gland carcinoma overexpress HER2, only 4% (3 of 70) of patients with ACC overexpress HER2 [72]. Treatment with trastuzumab is mostly reported for salivary gland carcinoma and may be dependent on whether the HER2 gene is amplified. In a phase II trial of patients with salivary gland carcinoma overexpressing HER2, only 1 partial response (1 of 14) was observed [73]. Complete responses for patients have been reported in case reports [74]. Promise has been shown combining trastuzumab and chemotherapy for salivary duct carcinoma, but further studies are needed to evaluate this approach in ACC in selected patients [75].

## CURRENT TREATMENTS FOR ADENOID CYSTIC CARCINOMA

### Cytotoxic chemotherapy

Due to its complicated clinical course and vague etiology, medical therapy is not standardized for ACC, yet radical excision and postop-radiation is mainly utilized for locoregional control for early stage disease. Specifically, there are no National Comprehensive Cancer Network recommendations regarding specific chemotherapy regimens [76]. In advanced stage, conventional chemotherapy regimens are still utilized as first-line therapy. Cisplatin and 5-FU or CAP (cisplatin, doxorubicin, and cyclophosphamide) regimens can be used for combination chemotherapy [77]. In one study, patients with advanced salivary gland malignancy treated with the CAP regimen achieved partial response (PR) or stable disease (SD) rates of 67% (8 out of 12 patients) [78]. Agents commonly given as monotherapy for treating ACC are cisplatin, mitoxantrone, epirubicin, vinorelbine, paclitaxel, and gemcitabine. However, few of these agents have shown efficacy [77, 79]. In a review by Laurie et al., which systematically assessed chemotherapy regimens based on their outcome, single agent mitoxantrone or vinorelbine were recommended as reasonable choices. Use of anthracycline alone or combined with cisplatin were also suggested [79]. Because of the low response rate of current chemotherapy, targeted therapies have also been investigated in clinical trials with the goal of improving patient outcomes.

More recently, one study examined gemcitabine monotherapy as a treatment regimen. No objective responses were reported with 38% of patients (8 of 21) having progression of disease (PD) and 52% of patients (11 of 21) having SD. The authors suggested that gemcitabine might not be an effective drug for ACC treatment, even though it was well tolerated by the patients [80].

### Experimental agents

Even though traditional chemotherapeutic agents are still considered first line therapy in advanced ACC, newly developed molecular targeting drugs have been explored. Due to its rarity, there are no disease specific agents designed for the treatment of ACC. Therefore, drugs that are currently used for more common cancers have been attempted on patients with ACC based on molecular findings, targeting the same markers identified in other cancers. Most of these agents have been used in phase I or II clinical trials.

Given that c-kit overexpression is common in ACC cells, imatinib and dasatinib were investigated in several studies. One study added cisplatin after imatinib monotherapy and produced a better objective response rate (ORR, 3 of 28, 11% of the patients achieved PR) [53]. Imatinib or dasatinib monotherapy showed no objective responses [50–52, 54]. Among the patients enrolled in five clinical trials, 52 patients had SD, which consisted of 53% of total patients (52 of 98). In particular, a single-arm, planned two-stage, phase II trial by Hotte *et al*., evaluated imatinib treatment in ACC patients expressing c-kit [51]. However, the study was stopped after the first stage after no objective responses were observed. The authors hypothesized that these patients may upregulate wild-type c-kit and not genetically altered c-kit. While patients with exon 9 or 11 c-kit mutations responded well to imatinib in gastrointestinal stromal tumors, similar mutations were not present in ACC patients with most upregulating wild type c-kit [46, 81, 82]. Therefore, it appears that c-kit does not play a critical role in ACC tumorigenesis despite its overexpression.

Drugs targeting EGFR or its tyrosine kinase also have been used for the treatment of ACC. These include cetuximab, gefitinib, and lapatinib [58, 83–86]. Using gefitinib or lapatinib alone did not produce an objective response, but 79% of patients in the lapatinib study had SD and 36% had prolonged tumor stabilization of greater than or equal to 6 months [83, 84]. In another study of patients with known EGFR positivity, patients were classified by their clinical stage and different regimens were utilized, but cetuximab and cisplatin were given to all patients [58]. In this study, complete response (CR) (2 of 9 patients, 22%) was observed in the locally advanced, non-surgically amendable group, and other patients in the same group showed a PR of 22% (2 of 9 patients). Patients with distant metastasis showed 0 CR (0%, 0 of 12) and 5 PR (42%, 5 of 12). Compared with the gefitinib and lapatinib studies, a better ORR (>40% for all patients) was observed [58]. Better responses may have been related to patient selection, as possible lack of prior systemic chemotherapy was not reported in this study. Regardless, this is very promising because patient selection based on molecular status produced some objective responses.

Since it is unclear which pathway contributes most to the development of ACC, multi-targeted kinase inhibitors such as dovitinib, axitinib, sunitinib, sorafenib, and regorafenib have also been studied. One study with sunitinib reported no ORR [87] and three studies showed partial response rates around 10% (2 of 19, 10.5% for dovitinib, 3 of 33, 9% for axitinib, and 2 of 19, 10.5% for sorafenib, respectively) [88–90]. These medications share common molecular targets, such as VEGFR 2 and 3. However, it is uncertain whether blocking these shared targets or simultaneously attacking multiple targets played a critical role in tumor remission.

As mutations related to the PI3K pathway have been found in ACC, this pathway is also a potential therapeutic route. In line with this, everolimus, nelfinavir and MK-2206 have been tested in clinical trials [91, 92]. However, no objective responses were observed in studies with everolimus and nelfinavir. Results for the study using MK-2206 are not yet available. A possible explanation for the lack of clinical response could be that not every cell line possesses the genetic alteration in this pathway [9].

Based on preclinical studies, high expression of NF-κB in ACC cells supports the potential use of bortezomib. Two studies have been performed using bortezomib and doxorubicin as a combined regimen [93, 94]. One study reported 1 PR (1 of 10, 10%) with concurrent use of doxorubicin, but bortezomib alone did not display an objective response [94].

Abnormal activity of histone deacetylase was identified in several malignancies. It is known that HDAC functions as an oncogenic factor in tumor cells. Thus, inhibition of this protein presents antitumor activity [25, 26]. Vorinostat, which targets this pathway, was used in one clinical trial for ACC but only showed a 3% response rate (1 PR, 1 of 30 and 0 CR.) [95]. In addition, chidamide, a HDAC inhibitor with activity in the PI3K/Akt and MAPK/Ras signaling pathways, was used in a clinical trial in patients with advanced solid tumors and lymphomas, with 1 of 3 ACC patients exhibiting a PR [96, 97].

In summary, of the 19 clinical trials highlighted in Table 2, a total of 22 objective responses were achieved (22 of 397, 5.5%). SD was frequently observed (62%, 246 of total 397 patients). However, the slow-growing nature of ACC can be a confounding factor when assessing clinical endpoints such as SD and disease-control rate (DCR) given the often indolent course of ACC. Overall, the study with cetuximab and concurrent chemoradiation or chemotherapy showed the highest ORR (total 43%, 9.5% CR and 33% PR), but this regimen was only given to the EGFR positive patients [58]. Some of the regimens reached PR including the chidamide trial (33% PR, *n* = 3 patients) the imatinib and cisplatin combination (11% PR), dovitinib monotherapy (10.5% PR), sorafenib monotherapy (10.5% PR), bortezomib and doxorubicin combination (10% PR), axitinib monotherapy (9% PR), and vorinostat monotherapy (3% PR).

**Table 2:** Completed and ongoing clinical trials for treatment of advanced adenoid cystic carcinoma

Phase	Agent	Dosing schedule	Biologic target	Status	Primary endpoint	Result	Reference
II	Regorafenib (*n* = 38*)	Oral 120 mg daily for 3 weeks in a 4-week cycle	VEGFR1, VEGFR2, VEGFR3, KIT, RET, BRAF, FGFR1 [116]	Recruiting participants	PFS at least 6 months, ORR (CR + PR) for 6 months	Not reported	NCT02098538
I	Chidamide (CS055/HBI-8000) (*n* = 3^+^)	Oral doses ranging from 5–50 mg either twice or three times per week for 4 consecutive weeks every 6 weeks	Histone deacetylase (HDAC), Akt, mTOR, Raf, Erk1/2, p21, Cdk4 [97]	Completed	Safety, efficacy PR	-Well tolerated -PR: 1 of 3 patients (33%)	[96]
II	Vorinostat (*n* = 30)	Oral 400 mg daily for 4 weeks in a 4-week cycle	HDAC	Active, not recruiting	ORR up to 6 months	-PR: 1 patient with response duration of ≥ 11.2 months (3%). -SD: 25 patients (83%). -Median PFS: 12.7 months. -Median OS has not been reached. -6 patients (20%) showed PFS > 1 year.	NCT01175980 [95]
II	Sorafenib (*n* = 23)	Oral 400 mg 2 times daily in a continuous schedule	Serine/threonine kinases c-Raf/b-Raf, VEGFR2, VEGFR3, PDGFR- β, FMS-like tyrosine kinase 3, c-kit, p38α	Completed	-PFS at 12 months -Secondary endpoint: ORR, OS, toxicity	-PFS at 12 months: 46.2%. -Median PFS: 11.3 months. -Median OS: 19.6 months. -PR: 2 patients among 19 assessable patients (11%). -SD: 13 of 19 patients (68%). -PD: 4 of 19 patients (21%).	Eudra CT2008–000066- 22 [90]
II	Cetuximab (*n* = 23^+^)	400 mg/m^2^/week followed by 250 mg/m^2^/week until progression	EGFR	Completed	CR, PR, SD	No objective responses.SD: 20 of 23 patients (87%)	[86]
I, II	Cetuximab + Intensity modulated radiation therapy (IMRT) (*n* = 49*)	-7 days prior to RT: IV weekly cetuximab 400 mg/m^2^ body surface -After RT start: IV weekly cetuximab 250 mg/m^2^ body surface	EGFR	Recruiting participants	-Toxicity of the combined therapy composed of RT + cetuximab -Secondary endpoint: ORR, PFS, OS	Not reported.	NCT01192087 [85]
II	Cetuximab + RT + cisplatin or Cetuximab + cisplatin and 5-FU(*n* = 21^)	-Locally advanced ACC (*n* = 9): IV cetuximab loading dose 400 mg/m^2^ 1 week prior to radiation, followed by weekly IV cetuximab 250 mg/m^2^ + concomitant RT + weeks 1, 3, 5 IV cisplatin 75 mg/m^2^ on days 1, 21 and 42. -Metastatic ACC (*n* = 21): IV cetuximab loading dose 400 mg/m^2^ in the 1st week of treatment followed by weekly IV cetuximab 250 mg/m^2^ + IV cisplatin 75 mg/m^2^ on day 1 + continuous 5-FU IV 1000 mg/m^2^/day on days 1–4.	EGFR	Completed	-PFS -Secondary outcome : ORR, OS	-Locally advanced ACC (*n* = 9): Median PFS 64 months. 2 CR (22%), 2 PR (22%), 5 SD (55.6%), no PD (0%). OS was not reached. -Metastatic ACC (*n* = 12): Median PFS 13 months, Maximum PFS 48 months. 5 PR (42%), 7 SD (58%), no PD (0%). OS 24 months.	EudraCT 2006–001694-23 [58]
II	Dovitinib (*n* =20*)	Oral 500 mg daily for 5 days, 2 days off each week in a 4-week cycle	VEGFR1, VEGFR2, VEGFR3, FGFR1, FGFR2, FGFR3, PDGFR- β [117]	Active, not recruiting	ORR and SD.	Not reported	NCT01678105
II	Dovitinib (*n* = 21)	Oral 500 mg daily for 5 days, 2 days off each week in a 4-week cycle	VEGFR1, VEGFR2, VEGFR3, FGFR1, FGFR2, FGFR3, PDGFR- β [117]	Active, not recruiting	ORR	-PR: 2 of 19 evaluable patients (10.5%). -9 patients had SD >6 months (43%). -6 patients with shorter follow-up did not show progression. (total SD: 15) (71%) -4 patients (19%) progressed early < 4 months.	NCT01524692 [88]
II	Axitinib (*n* = 33)	Oral 5 mg 2 times daily in a 4-week cycle	VEGFR1, VRGFR2, VEGFR3, c-kit [118]	Active, not recruiting	ORR	-PR: 3 of 33 patients (9%). -25 patients had SD (76%), 11 (33%) had SD for ≥ 6 months.	NCT01558661 [89]
II	Bortezomib + doxorubicin (*n* = 24)	-Days 1, 4, 8 and 11 IV push bortezomib 1.3 mg/m^2^ in a 3-week cycle until progression -Days 1 and 8 IV doxorubicin 20 mg/m^2^ added at the time of progression	26S proteasome, NF-κB	Completed	ORR	-Bortezomib only: no objective response. 15 of 21 evaluable patients presented SD (71%), with median PFS 6.4 months and OS 21 months. -Doxorubicin added: 1 of 10 evaluable patients had PR (10%), 6 had SD (60%).	NCT00077428 [94]
II	Bortezomib + doxorubicin (*n* = 10*)	-Days 1, 4, 8 and 11 IV push bortezomib 1.3 mg/m^2^, with days 1 and 8 IV doxorubicin 20 mg/m^2^ in a 3-week cycle. After 14 cycles, weekly bortezomib alone 1.6 mg/m^2^ on days 1, 8 and 15 every 4 weeks if there is no progression.	26S proteasome, NF-κB	Completed	ORR, SD rate	Not reported	NCT00581360 [93]
II	Nelfinavir (*n* = 15)	Oral 1250 mg 2 times daily	MAPK, PI3K/Akt signaling pathway	Completed	PFS, ORR	-No objective responses. -SD: 7 patients (47%) (at some point during trial), 2 of them had SD ≥ 6 months (13%). -PD: 9 patients among 12 assessable patients (75%). -Median PFS: 5.5 months.	NCT01065844 [91]
II	Sunitinib (*n* = 14)	Oral 37.5 mg daily in a 4-week cycle	VEGFR1, VEGFR2, VEGFR3, c-kit, PDGFR-α, PDGFR- β, RET, FLT3	Completed	ORR	-No objective responses. -SD: 11 patients (79%).-PD: 2 patients (14%). -Median OS: 18.7 months. -Median time to progression: 7.2 months.	NCT00886132 [87]
II	MK 2206 (*n* = 19*)	MK 2206 oral once weekly for 4 weeks in a 4-week cycle	Akt	Active, not recruiting	ORR	Not reported	NCT01604772
II	Gemcitabine (*n* = 21)	Days 1 and 8 IV 1250 mg/m^2^ in a 3-week cycle	Nonspecific	Completed	ORR	-No objective responses. -11 patients had SD (52%), 10 of them had SD (48%) for ≥ 6 months. -8 patients showed PD after 4 cycles (38%).	NCT00017498 [80]
I	Imatinib (*n* = 4)	Oral 400 mg daily	c-kit	Completed	OR, SD	-No objective responses. -1 patient with SD (25%)	[50]
II	Imatinib (*n* = 16)	Oral 400 mg twice per day	c-kit	Completed	OR, SD	No objective responses. -9 patients with SD (56%)	[51]
II	Imatinib (*n* = 10)	Oral 400 mg daily, dose modification allowed.	c-kit	Completed	ORR	-No objective responses. -2 patients (20%) had SD, both for ≥ 6 months.	[52]
II	Imatinib + cisplatin (*n* = 28)	Oral imatinib 800 mg daily for 2 months, followed by cisplatin IV 80 mg/m^2^ every 4 weeks with Oral imatinib 400 mg daily up to 6 cycles. Oral imatinib 400 mg daily after completion of chemotherapy.	c-kit	Completed	ORR^#^	-PR: 3 patients (11%). -SD: 19 patients (68%). -Median time to progression : 15 months-Median OS: 35 months.	[53]
II	Dasatinib (*n* = 40^+^)	Oral 70 mg daily for 4 weeks in a 4-week cycle.	c-kit	Active, not recruiting	ORR, PFS	-No objective responses. -0 patient had PR (0%), 21 patients had SD (52%). -Median PFS was 4.8 months.	NCT00859937 [54]
II	Lapatinib (*n* = 19^+^)	Oral once daily for 4 weeks in a 4-week cycle.	EGFR, erbB2(HER2)	Completed	ORR	-No objective responses. -15 patients had SD (79%). 9 of them had SD for ≥ 6 months (47%).	NCT00095563 [83]
II	Gefitinib (*n* = 18^+^)	Oral 250 mg daily	EGFR	Completed	ORR	-No objective responses. -7 patients (38%) had SD for ≥ 9 months. -Median PFS: 4.3 months. -Median OS: 25.9 months.	[84]
II	Everolimus (*n* = 34)	Oral 10 mg daily in a 4-week cycle	mTOR	Completed	-PFS rate at 4 months. -Secondary endpoint: ORR	-4 month PFS probability was 65.5% but was not significantly differ from the null hypothesis (*p* = 0.076). -No objective responses.-27 patients had SD (79%), 13 of them had SD for ≥ 6 months(38%).	NCT01152840 [92]

* Estimated enrollment numbers may vary from the actual participant numbers.

+ This study includes not only ACC patients, but also non-ACC patients. ‘n’ here only represents the number of ACC patients among all the participants.

^ This study has 2 cohorts: locally advanced and metastatic ACC. All patients had EGFR-overexpressing ACC.

# Except this study, all the other studies used RECIST v1.0 or v1.1 criteria for the evaluation of the responses.

-ORR: objective response rate; PFS: progression-free survival; OS: overall survival; CR: complete response; PR: partial response; SD: stable disease; PD: progressive disease.

-NCT: this is the individual trial number registered in http://ClinicalTrials.gov, which is the website developed by cooperation of the U.S. National Institute of Health (NIH) and the Food and Drug Administration (FDA).

-EudraCT (European Union Drug Regulating Authorities Clinical Trials): this is the electronic European clinical trial database supported by European Commission (EC). All clinical trials done in European Union (EU) will be given their own EudraCT numbers.

-All studies were non-randomized.

### Cancer immunotherapy

Cancer immunotherapy represents the newest armamentarium in cancer treatment. Cancer immunotherapy can be classified into 3 major groups. Active immunization using anti-tumor vaccines to induce and recruit T cells, passive immunization based on monoclonal antibodies, and adoptive cell transfer to expand tumor-reactive autologous T cells *ex vivo* and then reintroduce these cells into the same individual [98, 99]. In ACC, cancer vaccination and adoptive immunotherapy using lymphocyte activated killer (LAK) cells and cytokines have been tried in a small number of clinical trials.

An *in vitro* study of adoptive immunotherapy for an ACC cell line was performed in 1996 by a Chinese group [100]. This group investigated the susceptibility of ACC cells to LAK cells under the influence of cytokines including tumor necrosis factor-α (TNF-α) and interferon-γ (IFN-γ). This study confirmed that LAK cells showed cytotoxicity against ACC cells. Also, the authors concluded that both TNF-α and IFN-γ could enhance this cytotoxic process. Previously, it was reported that these cytokines induced tumor differentiation and apoptosis [101, 102].

In line with this, a clinical trial using adoptive immunotherapy with chemoradiation was performed on two maxillary ACC patients in Japan [103]. Both patients were given radiation (2 Gy 5 times a week), 5-FU (IV, 250 mg 4 times a week) and peplomycin (subcutaneous injection, 5 mg once every 2 weeks) followed by intra-arterial injection of LAK cells, interleukin-2 (IL-2) and IFN-γ on day 7. Two weeks after the initiation of treatment, significant tumor regression was observed. Three weeks after finishing the treatment, a CT scan revealed tumor reduction with new bone formation of the sinus walls in both patients. Furthermore, the patients had a good quality of life, indicating that this regimen was well tolerated. Even though the mechanism was not fully understood, it is likely that cytokine-induced cell apoptosis and the cytotoxic effect of the LAK cells contributed to tumor regression. This study is promising, but given the very small sample size necessitates further trials to confirm its findings.

Cancer vaccination, another immunologic approach targeting specific cancer antigens, has also been attempted in ACC [104, 105]. A hypothesis regarding Cancer/testis antigens (CTAs) as a potential target of immunotherapy was suggested based on the finding that CTAs were highly expressed in a subset of ACC cells, but not in normal cells. In addition, it was speculated that the expression of CTAs was augmented by 5-aza-2′-deoxycytidine (5-aza-CdR), meaning that CTAs could become a more effective target antigen under the influence of 5-aza-CdR [105].

Similar to CTAs, WT1 (Wilm's tumor 1) antibodies and WT1-specific cytotoxic T cells were observed in many cancer patients, suggesting WT1 protein could be a target of cancer vaccination [104]. In a phase I clinical trial performed in Japan, WT1 peptide vaccination was used on ACC patients with pulmonary metastasis. The vaccine was given via intradermal injection, 3 mg each time, a total of 50 times on a weekly basis. Significant suppression of the tumor growth was shown during the one year of treatment, while rapid tumor growth and new metastases occurred after the withdrawal of therapy. This trial implicated the efficacy of WT1 vaccine in the treatment of ACC [106].

Immune checkpoint blockade is another potential therapeutic strategy for patients with ACC. Effective disease control with CTLA-4 and PD-1/PD-L1 inhibitors (e.g. ipilimumab, nivolumab) was observed in several types of cancer including melanoma, non-small cell lung cancer, renal cell carcinoma, and urothelial bladder carcinoma, among others [107, 108]. Data are limited for ACC patients, but enrollment is underway in trials accepting a variety of solid tumor patients. Clearly, more research and well-designed clinical trials using immunotherapy in ACC patients are needed, but very early studies have shown promise.

## CONCLUSION

Effective treatment to prevent indolent ACC from evolving into recurrent, metastatic disease remains a challenge. The recent whole genome sequencing of ACC holds exciting potential for better understanding molecular changes to facilitate targeted genetic therapies for patients.

The molecular finding of the *MYB-NFIB* fusion gene has the greatest potential to target what appears to be a fundamental event in disease pathogenesis. In addition, whole exome and genomic sequencing revealed other genetic alterations in the *MYB* pathway in about 70% of ACC samples. Currently, no drugs are available to target the *MYB* pathway. Therefore, every effort should be made to develop novel agents targeting the chimeric fusion gene and *MYB* pathway. The most cost effective approach is to start *in vitro*, but establishing a validated ACC cell line has been challenging [109]. However, a recent study holds promise for immortalizing a primary ACC cell line with both epithelial-myoepithelial cells and some phenotypic molecular features of ACC [110]. Further studies are warranted to better confirm and characterize this cell line. In addition, xenograft mouse models of ACC have been developed and require further validation [111] These are critical tools for high throughput early drug testing and studies to better understand disease pathogenesis.

In addition, despite ACC being rare, genome and exome sequencing studies searching for driver genes have demonstrated that fundamental pathways in other cancers are shared in ACC. In particular, the second most common pathway in terms of frequency of genetic mutations was related to chromatic remodeling. Since histone pathology is significantly involved in this pathway, newly developed agents targeting histone-related biomarkers warrant further investigation. For example, romidepsin is a HDAC inhibitor currently approved for the treatment of cutaneous and peripheral T cell lymphoma [112]. This agent has potential for ACC as well and should be studied in future clinical trials. As several other pathways are also implicated in ACC, stratifying patients based on molecular and histologic subtypes will be critical in order to better understand clinical-pathologic-molecular correlations with different subtypes. In addition, it is unclear whether these shared pathways hold promise for utilizing existing agents in combination, which may suggest that downstream fundamental pathways associated with proliferation, cell cycle regulation, angiogenesis, and cell adhesion could also hold promise. Alternatively, other mutations in ACC are less prevalent in other cancers (e.g., *SMARCA2*), which if confirmed by additional studies warrant further investigation as drug targets in ACC.

Overexpression of key proteins has also been demonstrated in ACC, including c-kit, EGFR, SOX, VEGF, and AQP1. Using monoclonal antibodies to target these pathways has been attempted in clinical trials. Most notably, using cetuximab in EGFR-positive ACC patients resulted in relatively high ORR. This suggests that patient selection based on molecular findings can guide therapy choice. Other newly found proteins that were overexpressed in ACC could be novel molecular targets as well. Better clinical trials stratifying patient response to therapy based on molecular and histologic subtypes will be critical to assess which subtypes of patients respond. Also, given the cellular bipolarity in ACC and different biomarkers from those cells, simultaneous use of different types of targeted agents might be another therapeutic strategy.

Immunotherapy trials for ACC are in their infancy. The findings by Stephens *et al*. and Ho *et al*. that ACC tumors carry relatively few mutations (means = 13 and 22, respectively) compared to other solid tumors has therapeutic implications [9, 22, 113]. This may indicate that some types of immunotherapy, such as CTLA-4 and PD-1/PD-L1 blockade as monotherapy, may not be as effective as other cancers with higher mutational loads (e.g., melanoma and non-small cell lung cancer) that tend to respond better.

Recent randomized data indicate that combination therapy with nivolumab and ipilimumab was superior to ipilimumab monotherapy in patients with untreated melanoma [114]. In some cases, dramatic clinical responses can be seen after only one dose [115]. Utilizing a similar approach with combination trials for immunotherapy using dual immune checkpoint blockade or combining immune checkpoint blockade with vaccines or adoptive T cells should be investigated. In addition, the relatively small number of mutations in ACC tumors suggest potential for targeted therapies as well. The fact that each downstream mutation from *MYB-NFIB* is relatively rare across various ACC tumors further reinforces the critical need for individualized tumor phenotyping.

Given its indolent course, more effective treatments for ACC hold exciting potential with the goal of long-term management and avoiding recurrence and development of metastatic disease. Given its rarity, ACC has been understudied compared to more common cancers. To date, few objective responses have been observed in clinical trials. In addition, drug development has been challenging given the slow-growing nature of ACC often confounding SD reported in trials, the variability of historical data, and the lack of randomized control trials. The combination of a high fidelity chimeric fusion protein and a number of well validated genes and proteins that are common to other malignancies hold promise for better designs of therapeutic studies in ACC. This genomic blueprint should lead to future studies combining targeted, epigenetic, and immunotherapy agents for patients with ACC. Through better understanding of disease pathogenesis and development of targeted therapeutics, the goal is to improve outcomes for patients with ACC.
